# Virtual reality for assessing emergency medical competencies in junior doctors – a pilot study

**DOI:** 10.1186/s12245-024-00721-2

**Published:** 2024-09-27

**Authors:** Franca Keicher, Joy Backhaus, Sarah König, Tobias Mühling

**Affiliations:** 1 Institute of Medical Teaching and Medical Education Research, University Hospital Würzburg, Würzburg, Bavaria Germany; 2 University Hospital Würzburg, Children’s Hospital, Würzburg, Bavaria Germany

**Keywords:** Virtual reality, Emergency medical competencies, Competence assessment, Junior doctors, Clinical reasoning

## Abstract

**Background:**

The teaching and assessment of clinical-practical skills in medical education face challenges in adequately preparing students for professional practice, especially in handling emergency situations. This study aimed to evaluate the emergency medical competencies of junior doctors using Virtual Reality (VR)-based scenarios to determine their preparedness for real-world clinical situations.

**Methods:**

Junior doctors with 0–6 months of professional experience participated in one of three VR-based emergency scenarios. These scenarios were designed to test competencies in emergency medical care. Performance was automatically assessed through a scenario-specific checklist, and participants also completed self-assessments and a clinical reasoning ability test using the Post-Encounter Form.

**Results:**

Twenty-one junior doctors participated in the study. Results showed that while general stabilization tasks were performed well, there were notable deficiencies in disease-specific diagnostic and therapeutic actions. On average, 65.6% of the required actions were performed correctly, with no significant variance between different scenarios. Participants achieved an average score of 80.5% in the Post-Encounter-Form, indicating a robust ability to handle diagnostic decisions. Self-assessments did not correlate significantly with objective measures of competency, highlighting the subjective nature of self-evaluation.

**Conclusion:**

VR-based simulations can provide a detailed picture of EMC, covering both diagnostic and therapeutic aspects. The findings of this pilot study suggest that while participants are generally well-prepared for routine tasks, more focus is needed on complex case management. VR assessments could be a promising tool for evaluating the readiness of new medical professionals for clinical practice.

**Supplementary Information:**

The online version contains supplementary material available at 10.1186/s12245-024-00721-2.

## Background

The conveyance of clinical-practical skills constitutes a core principle within contemporary medical education curricula. Nevertheless, there exists a discrepancy between the efforts to teach or assess competencies and the significant challenges encountered in medical professional practice [1–3]. Handling emergency situations, which demand clinical decision-making under time pressure, poses a particular challenge for both medical students and junior doctors [4, 5]. In both scenarios involving standardized simulated patients and workplace-based assessment, students performed significantly worse in emergency situations compared to routine tasks [6, 7]. Please confirm if the section headings are correctly identified.Section headings are correctly identified.

To address this gap in emergency medical competencies (EMC), simulation environments implemented in virtual reality (VR) represent a promising approach. Moreover, efforts have been directed towards leveraging the technical capabilities of VR simulations for practical examinations, particularly in settings like objective structured clinical examinations (OSCEs) [8–10]. VR simulations offer highly standardized scenarios, also providing features such as real-time adjustment of difficulty levels and automatic performance evaluation [11–13]. Unlike examinations using physical, pre-defined models, VR-based examination scenarios can be easily adapted [14], for example, to prevent examinees from sharing relevant information with each other. Although the initial development costs of VR scenarios are high, they are likely to be amortized with frequency use [14]. However, this issue requires further clarification, particularly in the context of VR-based assessments. Furthermore, VR simulations show great potential for assessing overarching competencies such as clinical reasoning ability (CRA), because they allow real-time assessment in the execution of the clinical tasks. To date, written methods including multiple-choice questions with key feature cases and open-ended questions, such as the validated post-encounter form (PEF) have been employed to measure CRA through post-examination assessments with the candidates [15, 16]. Beyond curricular assessment for EMC or CRA, VR simulations could also serve as a structured tool for physicians prior to entering professional practice. By allowing for the demonstration of practical skills and decision-making in complex, real-world scenarios, they may provide valuable insights into practical skills that traditional performance parameters, such as final grades, do not capture.

Building on studies that have already assessed learners’ performance in various VR-based settings such as pediatric resuscitation training [11], fire in the operating room [12] and mass casualty incidents [13], our goal was to conduct a comprehensive assessment of EMC focusing on non-technical clinical skills through VR simulation. Unlike these examples, which depict rare situations, we selected three scenarios that junior doctors are likely to encounter frequently in clinical practice. Given the scarcity of objective data on EMC among doctors, identifying the nature and extent of potential deficits could inspire the development of future emergency medical curricula. In addition, by using the PEF as a traditional instrument to measure CRA, we aimed to determine if both methods measure a similar construct – namely, “clinical reasoning” – by correlation with EMC as measured through VR simulation. Since self-assessment is an easy and relatively effortless measure for assessing individual competencies, albeit with reported moderate accuracy [17], we wanted to determine how it correlates with objective performance in this specific context. In light of this, the present study aims to explore the following research questions:

1. Is the assessment of EMC using VR simulations feasible, and what outcomes can be achieved for junior doctors? Can different actions and levels of competency be made visible?
2. Is there a correlation between the VR simulation assessment and the outcomes of the CRA performance test?
3. Is there a correlation between the VR simulation assessment and the self-assessment of participants?

## Methods

### VR simulation

STEP-VR (version 0.13b) was used as the VR simulation of complex emergencies, co-developed with ThreeDee GmbH (Munich, Germany). The VR hardware setup for this study included a Schenker XMG Core 15 Laptop (chipset: Intel Core i7-9750 H, 6 × 2.6 GHz; graphics adapter: Nvidia GeForce GTX 1650, 4 GB GDDR6 VRAM) and an Oculus Rift S VR head-mounted display (HMD). The equipment enabled STEP-VR to run at a constant framerate of over 60 frames per second on “high quality” display settings of the HMD.

### Study design and measures

The study was conducted at a medical faculty in Germany from February to June 2023. Junior doctors with up to six months of professional experience at the University Hospital Würzburg were recruited. The study procedure and data protection details were explained to the participants, who then provided written consent. Demographic parameters and participants’ characteristics (age, gender, and prior experience with digital 3D and VR applications) were collected. Participants also completed a self-assessment questionnaire comprising 16 items, each addressing their agreement with different aspects of EMC. The design of this questionnaire was inspired by previous work on junior doctors’ preparedness in terms of clinical knowledge and skills [18]. Items representing overarching abilities, e.g. time management and prioritization of tasks, were incorporated.

**Fig. 1:**
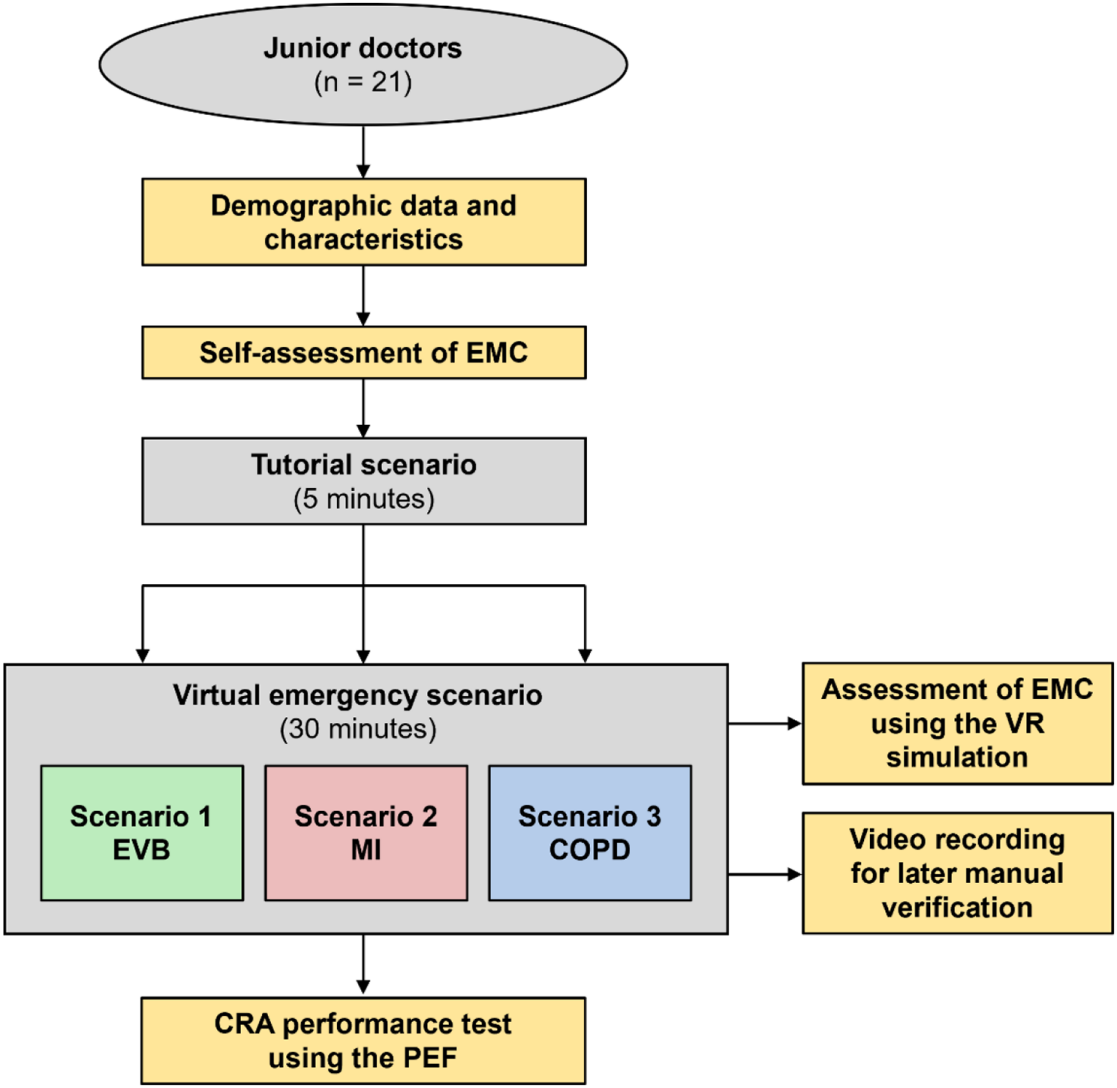
Overview of the data collection process. COPD: exacerbated chronic obstructive pulmonary disease, CRA: clinical reasoning ability, EMC: emergency medical competencies, EVB: esophageal variceal bleeding, MI: myocardial infarction, PEF: post encounter form

Before entering the VR scenario, participants received a 5-minute tutorial. They were instructed on the technical use of the VR controllers and functionalities of the virtual emergency department, including the layout of rooms, through a standardized audio guide. Subsequently, participants were randomized to one of three virtual emergency scenarios: (1) esophageal variceal bleeding (EVB), (2) myocardial infarction with third-degree atrioventricular block (MI), and (3) severe exacerbated chronic obstructive pulmonary disease (COPD, which had been previously evaluated [19]). The scenarios focused on clinical reasoning for differential diagnosis and initial therapy, gathering (menu-based) medical history, laboratory diagnostics, medical imaging (ultrasound / X-ray / computed tomography), emergency medications, ventilation therapy, and indication for interventional and surgical procedures (e.g. coronary angiography, endoscopy, abdominal surgery). All medical content was based on current guidelines [20–22]. Participants worked on the scenarios on their own and did not receive any explicit feedback from tutors/supervisors. The simulation system calculated the physiological effects of interventions on respiratory, circulatory, and laboratory parameters (e.g., by transfusion of blood products) and these effects could be observed as implicit feedback on the patient’s condition, the vital signs, or through repeated laboratory testing (e.g. changes in hemoglobin levels). The process of data collection is depicted in Fig. 1.

Table 1 presents an overview of the assessment instruments. Following the VR simulation, participants completed the PEF through a digital survey [15]. The form consisted of 5 free-text items, comprising the essential steps in the process of diagnostic clinical reasoning. The scoring rubric was developed based on the scenario content; the performance of participants was assessed by comparing with model answers. Grading was conducted by one of the authors (FK) in a blinded manner. Additionally, assessment of EMC using the VR simulation was automatically conducted using the STEP-VR program, which recorded all relevant actions performed by the user in a scenario-specific checklist. The checklists for each scenario had been previously established by the authors based on guidelines from professional societies [20–22]. All checklist items are listed in Table 4. During the VR-based assessment, a video recording of the scenario was made to allow for later manual verification of the automatically recorded checklist. However, no discrepancies were found comparing the two methods.

**Table 1:** Assessment instruments

Instrument	Description of the measure	Source	Scales and designations
Demographic data and characteristics	Participants’ information on age, gender, previous experience with digital 3D and VR applications	None	4 Items, each as separate selection
Self-assessment of EMC	Questionnaire for EMC as a tool designed to allow participants to evaluate their own competency levels	Created by the authors inspired by [18]	16 items on a 5-point Likert Scale, participants rated their agreement ranging from 1 = strongly disagree to 5 = strongly agree
Assessment of EMC using the VR simulation	Completion of scenarios with performance outcomes automatically generated based on actions.	[19, 23]	Embedded checklist with 9 (EVB and MI) or 10 items (COPD). The results were scored as an overall percentage.
CRA performance test using the PEF	Post-encounter form as a performance test aimed at scoring the CRA	[15]	5 open-ended questions for participants: • summary statement (max. 1 point) • problem list (max. 3 points) • differential diagnosis (max. 3 points) • most likely diagnosis (max. 1 point) • supporting data (max. 2 points) The results were scored as an overall percentage.

**Table 2:** Demographic data of participants, as well as previous experience in 3D and VR applications

Parameter	*N*	%
*Gender*		
Female	12	57
Male	9	43
Diverse	0	0
*Age*	27.3 ± 2.1y	
*Frequency of use of digital 3D applications*		
Never	15	71
< 1x/month	3	14
1-3x/month	1	5
1-6x/week	2	10
Daily	0	0
*Total duration of previous use of VR applications*		
None	9	43
Less than 1 h	7	33
Between 1 and 5 h	5	24
Between 5 and 10 h	0	0
More than 10 h	0	0

### Statistical analyses

Descriptive statistics including mean and standard deviation (SD) were calculated for the results of all measurement instruments and presented in the format of mean ± SD. Differences between multiple groups were calculated using ANOVA, with respective effect sizes reported as eta squared (η^2^). Pearson correlations were calculated to capture relationships between the results of different measurement instruments. The calculations and generation of figures were performed using GraphPad Prism (Version 10.1.2). It should be noted that, due to small sample size, all results of this pilot study should be considered as exploratory trends rather than definitive inferential conclusions.

## Results

### Participant demographics and characteristics

A total of 21 junior doctors participated in the study. Table 2 depicts the details of participants. The gender distribution (57% female participants) and age distribution (mean age 27.3 ± 2.1 years) were representative of young medical trainees. Overall, there were no significant specific prior experiences with VR and 3D applications.

**Table 3:** Self-assessment results for various aspects of EMC on a 5-point likert scale for participants’ agreement, with values listed in descending order

Item	Mean	SD
I know the reference values for heart rate, blood pressure, respiratory rate, and body temperature.	4.33	0.97
I can interpret an ECG of a patient with an emergency medical condition.	4.19	0.75
I can assess a patient using the ABCDE scheme.	4.15	0.75
I can conduct a focused history taking in clinical emergency.	3.90	0.62
I can perform a focused physical examination in a clinical emergency.	3.86	0.65
I can recognize a critically ill patient.	3.81	0.75
I can request the most important laboratory parameters in a clinical emergency based on the clinical presentation of a patient.	3.71	0.46
I can interpret the laboratory findings in a patient with an emergency medical condition.	3.67	0.86
**Mean of all items**	**3.49**	**0.57**
I can correctly assess the indications for performing an X-ray in a patient with an emergency medical condition.	3.43	0.68
I can interpret X-rays of a patient with an emergency medical condition.	3.29	0.78
I can prioritize tasks in emergency situations according to importance.	3.24	0.83
I know the most important medications that must be administered in clinical emergencies.	3.19	0.98
I can correctly determine the indication for further diagnostic and therapeutic interventions in clinical emergencies (e.g., endoscopy, cardiac catheterization).	3.14	0.79
I have a good time management in treating patients with emergency medical conditions	2.95	0.74
I can perform and interpret a focused emergency ultrasound in a patient.	2.81	1.29
I know the dosages of the most important medications that must be administered in clinical emergencies.	2.19	0.98

### Self-assessment of EMC

Across all items, the mean agreement value for self-assessed EMC was 3.49 ± 0.57, with detailed results in Table 3. Participants demonstrated above-average agreement values in self-rated abilities for history taking (3.90 ± 0.62), physical examination (3.86 ± 0.65), requesting laboratory tests (3.71 ± 0.46), and interpreting electrocardiograms (4.19 ± 0.75). Below-average agreement was observed in self-assessed knowledge related to procedural techniques such as sonography (2.81 ± 1.29) and in overarching skills such as task prioritization (3.24 ± 0.83) and time management (2.95 ± 0.74). The least agreement was noted for dosing of emergency medications (2.19 ± 0.98).

**Table 4:** Medical actions to be performed during assessment of the EMC using the VR simulation, which served as the basis for calculating the percentage performance. Indication of the proportion of participants (out of total *N* = 7) who correctly executed the action. IV: intravenous, ECG: electrocardiogram, CK: creatine kinase

Actions: Case 1 – EVB	*N*	%
Indication for gastroscopy	7	100
Successful hemodynamic stabilization (mean arterial pressure > 65 mmHg)	7	100
Collection of emergency laboratory tests (hemoglobin, coagulation, lactate)	7	100
Transfusion of packed red blood cells	6	86
Volume replacement with crystalloids	6	86
Administration of a prokinetic agent (IV erythromycin)	4	57
Administration of proton pump inhibitors in case of initially unclear bleeding source	3	43
Acute reduction of portal vein pressure (via vasoconstrictor)	2	29
Intravenous antibiotic therapy (covering gram-negative spectrum)	2	29
**Actions: Case 2 – MI**	**N**	**%**
Planning of primary percutaneous coronary intervention	7	100
Performance of a 12-lead ECG within 10 min	7	100
Collection of cardiac biomarkers (troponin, CK/CK-MB)	7	100
Administration of adequate analgesia	6	86
Administration of antiplatelet and anticoagulant therapy	5	71
Connection of an external pacemaker in symptomatic bradycardia	5	71
Medical treatment of symptomatic bradycardia	4	57
Administration of a second antiplatelet agent	1	14
Administration of antiemetic therapy for vegetative nausea	1	14
**Actions: Case 3 – COPD**	**N**	**%**
Performance of blood gas analysis	7	100
Suctioning of purulent sputum	6	86
Administration of broad-spectrum antibiotic therapy	5	71
Performance of microbiological diagnostics including sputum sample	5	71
Performance of a 12-lead ECG for differential diagnosis of cardiac origin	5	71
Indication for non-invasive ventilation in hypercapnic failure	4	57
Ordering of a chest X-ray examination	3	43
Symptomatic relief of dyspnea with morphine administration	3	43
Administration of bronchodilator therapy with ß2-mimetics/anticholinergics	2	29
Administration of anti-inflammatory therapy with prednisolone	1	14

### Assessment of EMC using the VR simulation

**Fig. 2:**
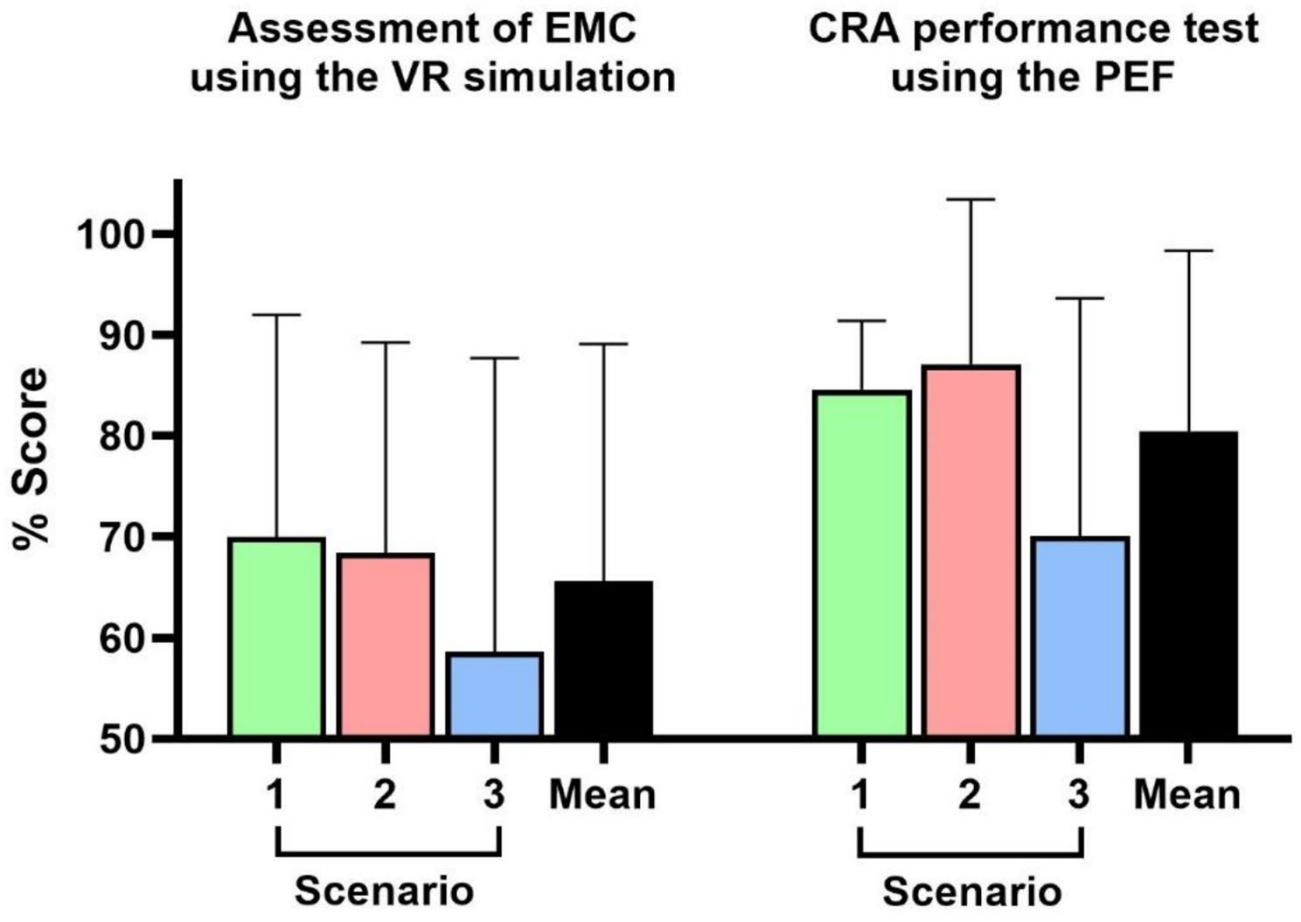
Percentage scores in the assessment of EMC using the VR simulation (left) and CRA performance test using PEF (right). The means and SD across the three scenarios, as well as total mean scores, are displayed

The assessment of EMC using the VR simulation was successfully conducted without technical issues for all 21 junior doctors (with 7 participants per scenario). On average, 65.6% ± 23.5% of the indicated medical actions were performed correctly across all scenarios. There were no significant differences between the scenarios (EVB 70.0% ± 22.0%, MI 68.4% ± 20.8%, COPD 58.6% ± 29.1%; η^2^ = 0.03; *p* = 0.76) (Fig. 2).

The analysis of individual actions revealed differences: Fundamental diagnostic procedures such as laboratory tests and general patient stabilization were accurately executed by nearly all participants. However, significant shortcomings were observed in performing case-specific diagnostics and therapy. For instance, in the scenario depicting EVB, only a small percentage of participants (29%) reduced portal vein pressure through vasoactive substances. In the scenario covering MI, administration of a second platelet aggregation inhibitor or antiemetic therapy for vegetative nausea was rarely performed (14% each). Additionally, the connection of an external pacemaker for severe, circulatory-effective bradycardia was also not consistently executed (57%). Similarly, only half of the junior doctors initiated non-invasive ventilation therapy for hypercapnic failure (57%), and systemic anti-inflammatory therapy for exacerbation of COPD was also rarely performed (14%). The internal consistency for all actions within each scenario was calculated, yielding a Cronbach’s α ranging from 0.74 to 0.84. The detailed results by action are presented in Table 4.

### CRA performance test using the PEF

Participant performance results using the PEF are depicted in Fig. 2. An average CRA score of 80.5% ± 17.8% was achieved. Notably, individual items of the PEF yielded similar results. Participants performed best in formulating possible differential diagnoses (82.5% ± 25.0%), but found it somewhat more challenging to decide on the correct diagnosis (76.2% ± 43.6%). Other items, such as creating a problem list (77.8% ± 24.3%) and naming supporting data for the most likely diagnosis (82.1% ± 21.1%), ranked in between.

While this study was only powered to detect very large effects, no such large differences were detected at *p* < 0.05 among the individual items or across the three scenarios regarding CRA measured by PEF (EVB 84.6% ± 6.8%, MI 87.14% ± 16.3%, COPD 70.1% ± 23.5%; η^2^ = 0.03; *p* = 0.16).

### Correlation of assessment measures and demographic data

**Fig. 3:**
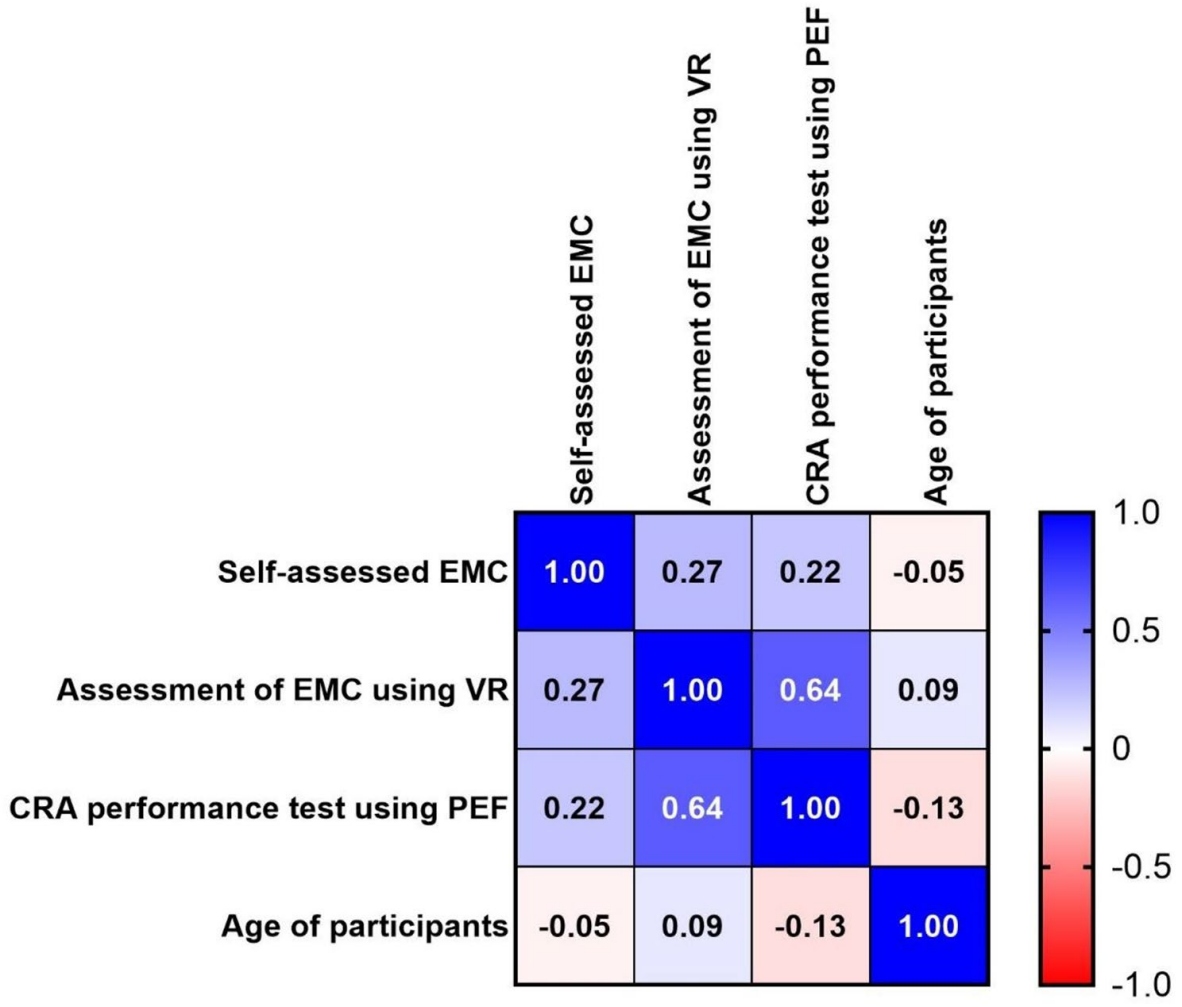
Correlation matrix of the different assessment measures and age of participants. The correlation coefficients (r) are displayed with color coding, indicating positive (blue) or negative (red) correlations

The assessment measures were correlated with each other as well as with the age of participants, which was the only ratio-scaled demographic attribute (Fig. 3). A strong and highly significant correlation (*r* = 0.64; *p* = 0.002) was found between the assessment of EMC using the VR simulation and the CRA performance test using the PEF. In contrast, a weak and non-significant correlation was found between the self-assessment of EMC and the assessment of EMC using the VR simulation as well as the CRA performance test, respectively (*r* = 0.27, *p* = 0.24 and *r* = 0.22, *p* = 0.35). Age was not associated with any of the measures. Similarly, other demographic data and characteristics which were nominally or ordinally scaled did not display any significant differences in group comparisons (not shown), at least to the extent assessable within the statistical power of the study.

## Discussion

The present study aimed to investigate the feasibility of using VR-based scenarios to evaluate the EMC skills of junior doctors. A representative sample of junior doctors was recruited, with their age and gender distribution mirroring that of the broader population of medical novices.

In the self-assessment for EMC, participants generally rated their abilities in history taking, physical examination, and diagnostic procedures (such as laboratory tests and ECG) as above average. However, deficits were primarily noted in therapeutic aspects and overarching skills (e.g. prioritization or time management), aligning with the focus of many current medical curricula and consistent with findings from previous studies [18]. Despite these plausible discrepancies in competency facets, there was no significant correlation between the self-assessment results in general and the outcomes from the EMC assessment using VR simulation or the CRA performance test using the PEF. This lack of correlation highlights that, although self-assessment is frequently used in clinical competency evaluations [18, 24, 25], it tends to reflect personal motivation and satisfaction with educational experiences [26], rather than providing an objective measure. Therefore, self-assessment alone is insufficient for evaluating competencies, but should be complemented by objective measures.

The assessment of EMC using VR simulation revealed that ~ 66% of the indicated medical actions were performed correctly. It is important to note that the actions were not weighted by the authors (‘life-saving’ actions were equally valued in the checklist alongside ‘supplementary’ medical actions), thus requiring further interpretation: Indeed, most junior doctors were successful in the correct selection of diagnostic measures and stabilizing patients in terms of circulation. However, significant deficiencies were observed in specific actions related to disease management, including critical measures like initiation of non-invasive ventilation. This is an important finding, as it suggests that such deficiencies may not be adequately captured by traditional final examinations (written or oral). VR-based assessments can thus provide valuable insights, particularly regarding practical competencies, which can stimulate curriculum development.

At first glance, participants’ CRA scores measured by the PEF were higher than those from the VR-based assessment of EMC. This difference should be interpreted with caution, as the PEF focuses exclusively on the diagnostic process, resulting in different items for each modality. However, this also aligns with the results of VR-based assessment of EMC, where participants performed better on the diagnostic items. Taken together, these results may suggest that junior doctors are relatively competent in diagnostics, but may need improvement in their therapeutic knowledge and decision-making.

Due to the lack of adequate objective data on EMC skills of graduates [4], comparing results is challenging. In a narrative interview study from the UK, 185 representatives of various levels of experience in clinical patient care agreed that graduates possess sufficient skills for diagnosing and treating typical clinical conditions. However, significant uncertainties were described when cases became more complex or those requiring emergency actions [27]. A more recent review, which primarily relies on the external assessment of supervisors, reached a similar albeit somewhat more heterogeneous conclusion regarding EMC among junior doctors [28]. Both review articles underscore the need to engage more frequently with complex clinical conditions and scenarios either during medical school or at the beginning of professional practice, an area which is currently lacking. VR-based learning environments offer optimal conditions for this purpose, as their complexity can be increased almost indefinitely [14]. This is particularly beneficial at the transition from education to further training, without the need for additional material and personnel.

Lastly, the present study demonstrated a strong correlation of assessment of EMC using VR simulation with CRA performance test using the PEF. This suggests that the VR-based scenarios and traditional assessment instruments, such as the PEF, demonstrate convergent validity in measuring the overarching construct of CRA. As a limitation, the PEF consists of items (open ended questions) that primarily focus on the diagnostic process. Further studies could explore the correlation of VR-based assessments and measurement tools that also cover the therapeutic process, such as the script concordance test [29]. Importantly, while the pilot study demonstrated relatively high internal consistency for the items of VR-based assessment and convergent validity for the construct CRA, other test quality criteria (such as discriminant validity or content validity) remain unaddressed. However, there is evidence from other studies supporting discriminant and content validity of VR-based approaches. For instance, the assessment of emergency medical skills using VR 360° videos was able to distinguish different levels of prior experience [10]. Additionally, a VR application for assessing the effectiveness of resuscitation measures was considered realistic and valid regarding the content by a larger group of experienced OSCE examiners [9]. We recently demonstrated that the difficulty of a VR-based OSCE station was comparable to an analog station, with even slightly superior discriminative power regarding an entire curricular OSCE [8]. Although further evidence on the validity and reliability of VR-based assessments would be beneficial, these platforms show promise for evaluating preparedness for real-world situations by providing replicas that users perceive as authentic. This can be particularly valuable in entry tests for junior doctors, ensuring that they possess the necessary skills and knowledge to effectively navigate complex clinical environments. Thus VR-based assessments could assist in identifying and addressing competency gaps, serving as an initial step towards enhancing patient care.

### Strengths

This study tested the utilization of VR-based complex emergency scenarios for competency assessment of junior doctors. An objective picture of EMC across three scenarios was obtained from a representative sample of graduates at the study site. Furthermore, the scenarios used have been employed in teaching since 2020 and have been continuously refined since then. Multiple measures, including the PEF as a validated tool, were used to demonstrate convergent validity in measuring the overarching competence of CRA.

### Limitations

A relatively small number of participants was recruited at only one site, limiting the generalizability of the results. Consequently, the study was only capable of identifying very large effects, serving primarily as an exploratory starting point for future research. However, it allowed for some plausible and statistically significant conclusions. The items for assessing performance in the VR scenarios used in this study were created based on guidelines and clinical experience by subject matter experts. However, they have not yet been analyzed for characteristics such as content or discriminant validity within a larger collective.

## Conclusions

The findings of this study confirm the feasibility of utilizing VR-simulation to assess EMC among junior doctors. The obtained results provide a detailed perspective on junior doctors’ ability to manage emergency medical situations. Despite the general proficiency in clinical reasoning and routine emergency tasks (such as patients stabilization) observed among participants, the study highlighted specific aspects, particularly in complex disease-specific diagnostics and management, where performance could be improved. VR-based scenarios may become a valuable tool for assessing clinical competencies in entry tests for junior doctors in the future.

## Electronic supplementary material

Below is the link to the electronic supplementary material.

Supplementary Material 1

## Data Availability

All data supporting the findings of this study are available in the electronic supplementary material accompanying this article.
